# Transmission analysis of a large tuberculosis outbreak in London: a mathematical modelling study using genomic data

**DOI:** 10.1099/mgen.0.000450

**Published:** 2020-11-11

**Authors:** Yuanwei Xu, Jessica E. Stockdale, Vijay Naidu, Hollie Hatherell, James Stimson, Helen R. Stagg, Ibrahim Abubakar, Caroline Colijn

**Affiliations:** ^1^​ Centre for Mathematics of Precision Healthcare, Department of Mathematics, Imperial College London, London, UK; ^2^​ Department of Mathematics, Simon Fraser University, Burnaby, BC V5A 1S6, Canada; ^3^​ University College London, London, UK; ^4^​ National Infection Service, Public Health England, London, UK; ^5^​ Usher Institute of Population Health Sciences and Informatics, University of Edinburgh, Edinburgh, UK; ^6^​ Institute for Global Health, University College London, London, UK

**Keywords:** genomic epidemiology, infectious disease, modelling, machine learning, tuberculosis

## Abstract

Outbreaks of tuberculosis (TB) – such as the large isoniazid-resistant outbreak centred on London, UK, which originated in 1995 – provide excellent opportunities to model transmission of this devastating disease. Transmission chains for TB are notoriously difficult to ascertain, but mathematical modelling approaches, combined with whole-genome sequencing data, have strong potential to contribute to transmission analyses. Using such data, we aimed to reconstruct transmission histories for the outbreak using a Bayesian approach, and to use machine-learning techniques with patient-level data to identify the key covariates associated with transmission. By using our transmission reconstruction method that accounts for phylogenetic uncertainty, we are able to identify 21 transmission events with reasonable confidence, 9 of which have zero SNP distance, and a maximum distance of 3. Patient age, alcohol abuse and history of homelessness were found to be the most important predictors of being credible TB transmitters.

## Data Summary

Raw data are available in the European Nucleotide Archive with accession number ERP003508. The beast xml file is provided in supplementary information for this article that can be found on Figshare (https://figshare.com/) at: 10.6084/m9.figshare.12413012.

**Impact Statement**

Improvements in sequencing technology have enabled rapid sequencing of pathogen genomes from infected individuals in an infectious disease outbreak. The high volumes of data generated, e.g. through whole-genome sequencing (WGS), have been used by researchers to help elucidate person-to-person transmission events. However, WGS data alone may not be sufficient to reconstruct transmission events, especially when there is a lack of variability in the sequences. Conversely, patient covariate data is a rich source of information for outbreak investigation. Hence, combining WGS with epidemiological data and patient covariates should yield improved understanding of transmission. In this paper, we explore retrospectively how sequence data, combined with epidemiological, clinical, demographic and patient behavioural data, can help improve our understanding of transmission events in a large tuberculosis outbreak in London, England, and to identify covariates that may contribute to transmission. We combine phylogenetic estimation, Bayesian transmission inference and machine-learning tools. Through this integrative analysis, we are able to identify more transmission events with reasonable confidence than previously studied, identify credible transmitters and associate transmission to covariates.

## Introduction

Analyses of chains of transmission – i.e. who infected whom – are critical tools within outbreak control. In tuberculosis (TB), transmission analysis is particularly challenging, because TB has the potential for dormancy in infected individuals many years after transmission, making it hard to distinguish recent transmission from reactivation. Additionally, in low-incidence, wealthier nations, the disease is often concentrated in populations that are under-served by traditional health-care models, resulting in infectious individuals taking many months to be diagnosed. Within the public-health process for patients with infectious respiratory disease, it can be challenging to identify an individual’s contacts over long periods of time, particularly within under-served population groups, who may have unstable housing and mistrust traditional systems of authority.

Since the advent of next-generation sequencing technologies has made this feasible [1], whole-genome sequencing (WGS) data are increasingly gathered in efforts towards TB control, and there have been high hopes that WGS technologies will greatly facilitate outbreak reconstruction. In high-income countries, WGS data and demographic and epidemiological data are now often gathered for TB. National TB control programmes following World Health Organization guidelines collect a standard set of data, including demographic and clinical data, along with data on treatment outcomes and bacteriology [2, 3], some of which are likely to be related to transmission. Local programmes may collect further variables, which can be crucial in controlling and eliminating outbreaks [3]. In 2017, England was the first country worldwide to roll out routine WGS for TB cases [4]. It is not clear to what extent WGS data will reveal transmission events, though it is now established that sequences alone, at least with current bioinformatics analysis pipelines, are insufficient to reliably determine precisely who infected whom [5–8]. But the context of increased availability of WGS data, together with demographic and clinical covariates, provides researchers with new challenges – to what extent can incorporating demographic and clinical data with WGS aid in understanding transmission?

Within London, particularly the north of the city, a long-standing outbreak of isoniazid (INH)-monoresistant TB, first defined by a shared RFLP cluster and later defined by a shared identical 24-loci MIRU-VNTR type (mycobacterial interspersed repetitive units-variable number tandem repeat type), has existed since 1995 [6, 9–11]. By 2013, there were 501 cases in total in the UK. Extensive contact tracing and transmission analysis were done in the first years following detection of the outbreak in 2000 [10, 11]. The outbreak has been of particular concern; there have been hundreds of cases and it has contributed to rising INH resistance in England [10]. The outbreak has showed signs of high transmissibility – with only brief contact sufficient for transmission [6, 11] – and a high proportion of smear-positive cases [6, 9]. During and after the outbreak, retrospective outbreak questionnaires and patient interviews were completed by TB clinic nurses, gathering information such as drug and alcohol use, history of homelessness or imprisonment, and treatment history. Recently, isolates from the outbreak cluster were sequenced with WGS to aid in resolving the transmission network, but due to low levels of detectable variation, individual transmission events could not be reliably inferred [6].

Here, we combine WGS data, data on times of sampling, and demographic, clinical and other host data to analyse this complex outbreak. We first reconstruct timed phylogenetic trees using WGS data together with sampling times. We introduce a new approach to reconstructing individual transmission events, jointly analysing a posterior collection of timed phylogenetic trees while sharing key model parameters. This takes phylogenetic uncertainty into account, while constraining reconstructed transmission events on different posterior phylogenies to have the same underlying epidemiological parameters. This analysis allows us to estimate how many unsampled cases there were, how long individuals took from original infection to infecting others, and the time between initial infection and sampling, taking phylogenetic and parameter uncertainty into account. Finally, we relate the extended demographic and clinical data to transmission by training machine-learning tools to predict which individuals were likely transmitters, using the covariate data alone.

## Methods

### Data

The London INH-resistant TB outbreak is characterized by Public Health England as cluster E1244 (strain type 424332431515321236423–52 and including an untypeable 3690 locus). Previous work documents the data collection, surveys and questionnaires, contact tracing and WGS used as part of outbreak investigations [6, 9–11]. The cluster was first identified using IS*6110* RFLP analysis, by a screening method based on PCR and then using a unique 24-locus MIRU-VNTR type [6]. In the work by Maguire *et al*. [9], cases were defined as part of the outbreak if the patients had an INH-monoresistant strain, were diagnosed between 1995 and 2006, had the RFLP or MIRU-VNTR pattern matching the outbreak, and were either a resident of London or had epidemiological links with London. The outbreak then continued after 2006 and was described with sequencing data by Casali *et al*. [6].

**Fig. 1.**
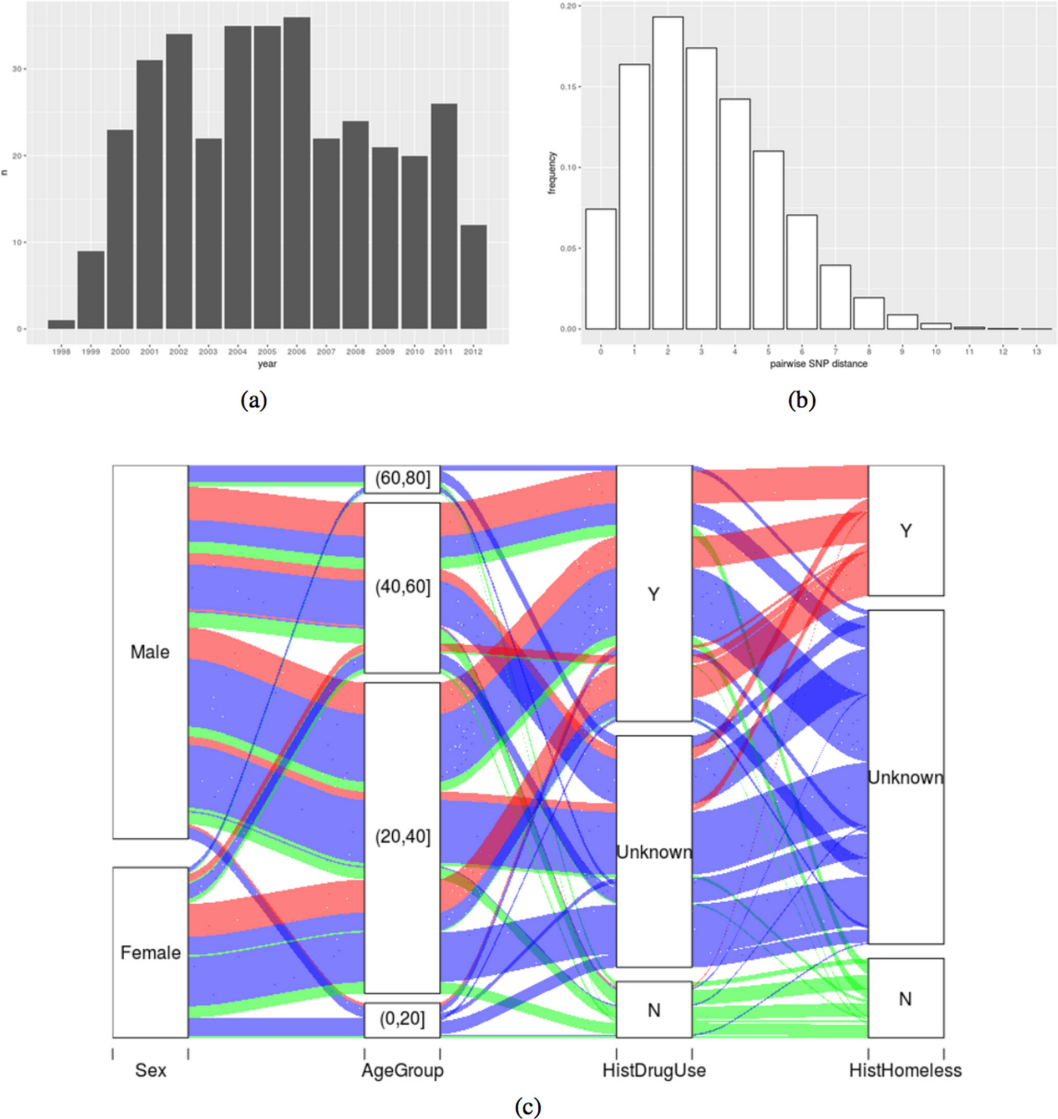
(a) Number of sequences in our dataset by year. (b) Frequency of pairwise SNP distance. (c) Illustration of some of the covariates in an alluvial plot showing how many individuals are in each category and how many share categories from one column to another. Colours correspond to homelessness history: Y (yes), red; N (no), green; unknown, blue. As an example of the interpretation of the plot, nearly all those who have a yes for a history of homelessness (red) either also have yes or unknown for a history of drug use (red bands reaching from Y and unknown in the ‘HistDrugUse’ column up to the Y category for ‘HistHomeless’).

Covariates (sex, age, region, born in the UK, occupation, ethnic group, sputum smear status, previous TB diagnosis, history of drug use, alcohol, presence of mental health concerns, homeless, history of homelessness, prison status and prison link) were obtained from the patient surveys, questionnaires and interviews, along with medical records; we visualize some of these data in Fig. 1(c). We took the following approach to missing data: for categorical variables with two strata (e.g. ‘yes’ and ‘no’; this describes most of our variables), if a variable was missing more than 40 % of its data, then the missing values were replaced by ‘unknown’. For all other variables, the R package Mice was used for multivariate imputation. In doing so, we had assumed that the missing data was missing at random. The decision to replace rather than impute the missing data is based on the observation that if many entries are missing, there may not be enough information for imputation, and so the result could be far from the truth. However, discarding the variable completely will result in a loss of information, and we wish to use the data that are available.

### SNP calling and phylogenetic reconstruction

Isolates were cultured and then whole-genome sequenced [6] on an Illumina HiSeq system with a read length of 100 bp at the Wellcome Trust Sanger Institute (Hinxton, UK); the raw data are available in the European Nucleotide Archive under the accession number ERP003508. Samples in this study were excluded from the analysis if any issues were recorded with their culturing in the lab, such as lack of growth, contamination or other reasons potentially impacting quality. An assessment of sequence quality was initially carried out using FastQC (version 0.11.2). Raw fastq reads were then filtered for length and trimmed for low-quality trailing base pairs using Trim Galore (version 0.4.1); any trimmed reads that were shorter than 70 bp were discarded. Reads were aligned to the H37Rv NC000962.3 reference genome using the BWA (Burrows-Wheeler Aligner - version 0.7.15) MEM (maximal exact match) algorithm, with duplicate reads removed using Picard’s (version 2.6.0) MarkDuplicates tool. SNPs in hypervariable regions, repeat regions and mobile elements were excluded. Local realignment round insertions and deletions (indels) was carried out using the gatk (version 3.6) IndelRealigner tool. SNPs were identified using FreeBayes (version 1.1.0) with a minimum mapping quality of 30 and minimum base quality score of 20. Isolates with a high proportion of apparent mixed or heterozygous SNP calls (i.e. those with more than 20 % reads supporting the reference allele) were excluded from the analysis. A variable-site alignment was created as a fasta-format multiple sequence alignment that excluded non-variant bases, along with an index mapping the base number of the alignment to the corresponding location on the reference genome. Calls made with a read depth of less than 30 across all the samples in the study were also excluded.

The phylogenetic tree-building software beast2 (version 2.6.1) [12] was used to build timed phylogenetic trees. A preliminary check using TempEst [13] showed positive correlation between genetic divergence and sampling time (Fig. S1, available with the online version of this article), and a moderate level of temporal signal (TempEst *R* ^*2*^=0.21). Because of moderate temporal signal in the SNP data, we adopted a strict molecular clock, supplying the tip dates, and we used a fixed rate parameter of 1.0×10*^−^* ^7^ per site per year, corresponding to 0.44 substitutions per genome per year [14]. We used a coalescent constant population model with a log-normal [0, 200] prior [15, 16] for the population size. Because the K3Pu model of nucleotide substitution was not available in beast2, we used the generalized time reversible (GTR) substitution model [17], which had the next lowest Bayesian information criterion (BIC) score (Δ6910.964) on the basis of model testing using iq-tree [18]. The GTR model with prior rates having a gamma distribution with rates in [0, ∞] and prior frequencies (estimated) in [0, 1] were applied, along with 0 proportion of invariant sites. We used the beast2 correction for ascertainment bias, specifying the number of invariant A, C, G and T sites as 758 511 1 449 901 1 444 524 758 336. Note that this must be manually added to the xml and may not appear when the xml is loaded into the BEAUti2 (version 2.6.1) software. We ran the Markov chain Monte Carlo (MCMC) method for 100 000 000 iterations, sampling every 10 000th iteration. We verified chain convergence (by confirming multiple independent chains converged to the same posterior values) as well as good mixing and an effective sample size (ESS) of greater than 200 for all parameters using Tracer (version 1.7.1) [19]. A maximum clade credibility (MCC) tree was created using TreeAnnotator (version 2.6.0) [20], with 10 % of the chain discarded as burn-in, resulting in a posterior collection of 9000 trees. Instead of trying to obtain a single optimal timed phylogenetic tree from this posterior set, we sampled a collection of 50 of them at random. This ensures that we capture as much diversity as possible from the beast posterior, to achieve robust uncertainty quantification in our subsequent analysis.

### Transmission inference

We performed Bayesian inference of transmission trees given a timed tree using the TransPhylo package in R [8], but we extended the approach to simultaneously infer transmissions on a subsample of beast trees rather than using just one. We based all downstream analysis on a combined set of posterior transmission trees inferred from these distinct phylogenetic trees. We also allowed the flexibility of sharing model parameters across different input phylogenetic trees, so that only a single parameter set is updated instead of *N* sets for *N* timed phylogenetic trees. This results in better mixing for the underlying MCMC algorithm than not sharing parameters.

**Fig. 2.**
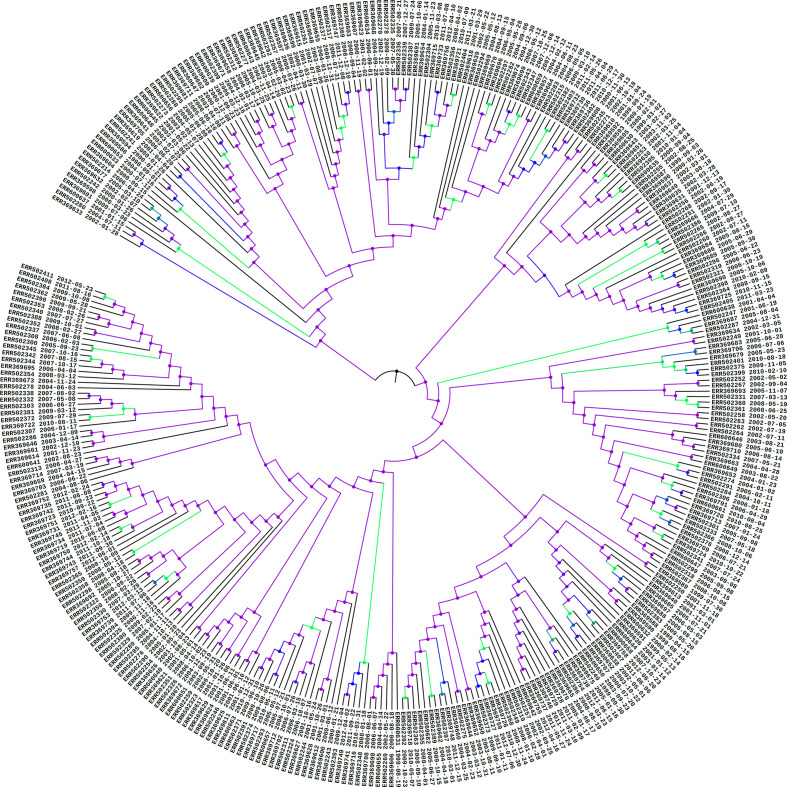
MCC tree of the beast analysis under a coalescent constant population model on a dataset consisting of 351 TB outbreak genomes sampled from patients in the UK. Branch colours correspond to different posterior probabilities: minimum, purple; midpoint, blue; maximum, green; undefined, black.

Since TransPhylo assumes that sequences are from unique hosts and is not designed to handle multiple sequences from the same host, in order to avoid confusion of a host infecting itself, we kept only the earliest sequence from each host in the input phylogenetic trees. A treespace [21] analysis did not suggest any multi-modality in the posterior beast trees (Fig. S2), but the posterior trees are discordant, with many nodes with low support (see Fig. 2). For this reason, rather than summarizing the posterior with just one MCC tree (as is standard), we opted to use a sample of 50 phylogenies to better capture phylogenetic uncertainty. We randomly sampled 50 trees from a subsample of the beast posterior, excluding the burn-in. This choice reflects a trade-off between TransPhylo computational burden and being representative of the full beast posterior. TransPhylo was run on the joint tree space of these 50 posterior phylogenetic trees, with parameter sharing, for 10^5^ iterations. Output transmission trees were collected every 50 iterations, to reduce correlation between subsequent trees in the sample. Simultaneous analysis on multiple phylogenetic trees, with parameter sharing between them, is a new addition to TransPhylo here. It allows the transmission inference to incorporate phylogenetic uncertainty, and unlike an analysis using TransPhylo separately on a set of input phylogenies, parameter sharing yields one estimate over 50 input trees as opposed to 50 estimates, one per input tree. The multi-tree capability used here is available in TransPhylo at github.com/xavierdidelot/TransPhylo [22].

The epidemiological generation time and time-to-sampling are both described by gamma densities with fixed parameters. For the generation time, the shape and scale parameters used are 1.3 (unitless) and 2.5 years, respectively; and for the time between infection and sampling, the shape and scale parameters used are 1.1 (unitless) and 6.0 years. These time quantities are known to be widely variable for TB outbreaks, but our parameter values were informed by previous TB outbreaks in well-resourced settings (for example [23, 24]). The offspring distribution in TransPhylo is a negative binomial distribution NB(*r, p*), with the second parameter, *p*, fixed to be 0.5. The mean is, therefore, equal to the first parameter, *r*, which is also the basic reproduction number *R*_0_. We fixed the within-host coalescent parameter *N_e_g* at 100/365. *N*_*e*_ *g* is the product of the coalescent in-host effective population size *N_e_* and the within-host generation time *g* [25]. The within-host generation time is a different parameter than the epidemiological (between-host) generation time. The epidemiological (between-host) generation time denotes the time between an individual becoming infected and infecting another, whereas the within-host generation time is the time between effective bacterial generations within a host. A prior belief that an effective approach to active case finding was implemented during this outbreak [26] was reflected in TransPhylo with an informative beta prior for the sampling probability denoted π, with the two parameters being eight and two. The date of the last sample was June 2012. TransPhylo was run with date T=∞, i.e. the finished outbreak scenario, because to our knowledge, cases matching the outbreak criteria were not identified subsequently.

### Patient-level prediction from metadata

The ‘ground truth’ answers to many questions in an outbreak reconstruction – who infected whom and when – are typically not known. We used the posterior transmission trees from TransPhylo as a proxy for this ground truth. For example, suppose that we are interested in predicting whether a host has transmitted TB. We can describe whether an individual is a ‘credible transmitter’ by setting a binary variable to be true if more than half of the posterior transmission trees suggest that the host infects at least one other; while the true transmission events are unknown this allows us to capture variation in the likelihood that an individual transmitted to another during the outbreak. We could also be more stringent by assigning a true label only when over 80 % of transmission trees imply that the host infects someone else; in this case, the resulting true positives will more closely reflect the TransPhylo estimates of who is a transmitter, but false negatives will likely increase as well.

Once we have extracted a host-level variable of interest from the posterior transmission trees produced by TransPhylo, we can then train a machine-learning algorithm to predict this target variable, using either (i) both the metadata and other predictors extracted from TransPhylo such as the generation time and time-to-sampling for each host, or (ii) the metadata alone. Here, we chose the latter because we are interested in assessing whether the covariates in the metadata have predictive power for identifying credible transmitters.

**Fig. 3.**
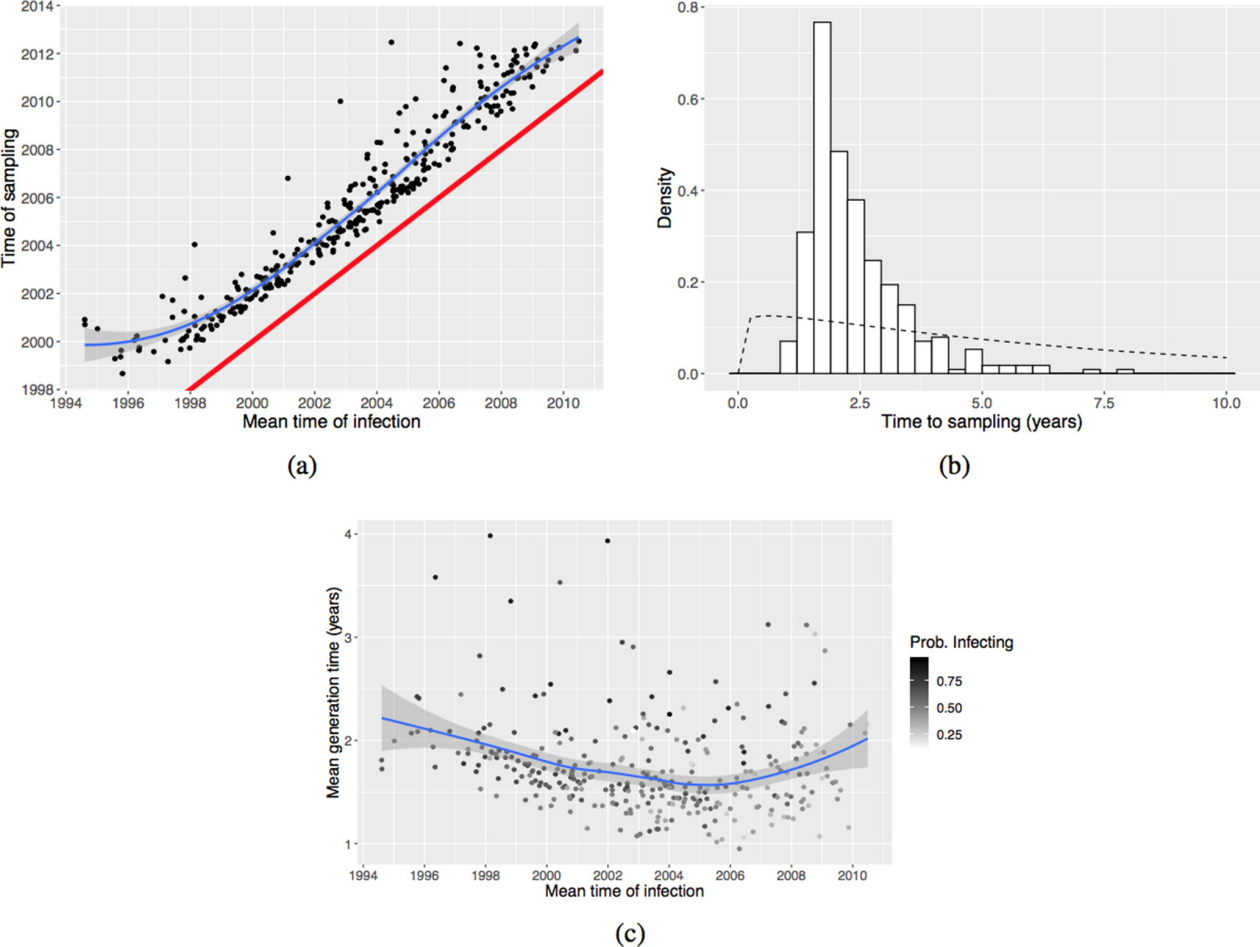
(a) Scatter plot of times of infection and sampling. Each dot corresponds to an individual host. A smooth line (blue) has been fitted, and a reference line (red) of *y=x* has been added to aid inspection. (b) Interval in years between times of infection and sampling in (a), overlaid with the prior gamma density used in TransPhylo (shape 1.1 and scale 6 years; dashed line). The few cases in the upper tail of the histogram correspond to cases earlier in the outbreak when sampling was poor. (c) Scatter plot of time of infection and generation time in years, each dot corresponds to an individual host. The individual cases are coloured by their probability of infecting others, with darker colour indicating a higher probability. In (a) and (c), a smoother has been fitted in order to better see the relation between the variables, using local polynomial regression fitting, or ‘loess’; the shaded area indicates 0.95 confidence interval level.

We explored two machine-learning tasks: predicting whether an individual is (likely) a transmitter of TB, and predicting whether an individual is estimated to have a longer-than-usual generation time. Accordingly, in the first task, the response was chosen to be a binary variable that is true if the posterior probability that the individual in question infects at least one other individual is greater than 0.5, and false otherwise. A random forest classifier was trained with fivefold cross validation, so that each model was used to predict data that it had not seen during training. In the second task, we created a new binary variable (‘long’ generation time or not). The generation time was estimated from the TransPhylo posterior transmission trees by subtracting the mean infection time of a host from the mean first transmission time of that host (Fig. 3c). The mean was taken over all posterior transmission trees in which the host ever infects another (regardless of who they infect, and even if they have low posterior probability of infecting anyone). Naturally, generation times are censored by the end time of the data; individuals infected very recently have had less opportunity to infect others, and any secondary cases we do have in the data will have happened rapidly. We considered the generation time to be long if it was greater than 2 years, and short otherwise. We trained a random forest classifier as in the first task, using the same set of training and validation data. Using 0.5 as a threshold of probability of transmission, TransPhylo predicted that 205 (62 %) cases were transmitters and 124 (38 %) cases were non-transmitters. A total of 64 (19 %) cases had generation times above 2 years and 265 (81 %) below 2 years.

## Results

beast2 analysis of 351 genomes resulted in an estimated substitution rate of 6.603×10^−8^ (95 % highest posterior density (HPD) 5.546–7.745×10^−8^) substitutions per site per year and an estimated time of the most recent common ancestor (tMRCA) of 1989 (95 % HPD 1986 to 1991), with ESS scores of 1131.9 and 2621.2 on the MCC tree, respectively. The MCC tree generated under a coalescent constant population model is shown in Fig. 2.

Of the 351 sequences in the final dataset, 94 were identical (that is, they were the same one sequence); Casali *et al*. [6] also found a high number of identical sequences. We used 329 of the sequences, among which there were 269 variable sites, for the transmission analysis. This restriction was because some individuals had multiple isolates in the data, and the TransPhylo model assumes that each tip in the phylogeny corresponds to one host. Accordingly, we used only the earliest sequence from each host. In Fig. 1(a), we show the total number of sequenced isolates per year between 1998 and 2012. The frequency of all pairwise SNP distances is shown in Fig. 1(b). Among other patterns, we note that if a patient had a history of homelessness, then they tended to also have used drugs; most patients were between age 20 and 40, with more males than females (Fig. 1c). There are missing data, which is to be expected, as patients may be unwilling to disclose some of this information, and record-keeping over a long period across multiple sites can be prone to error and loss.

**Fig. 4.**
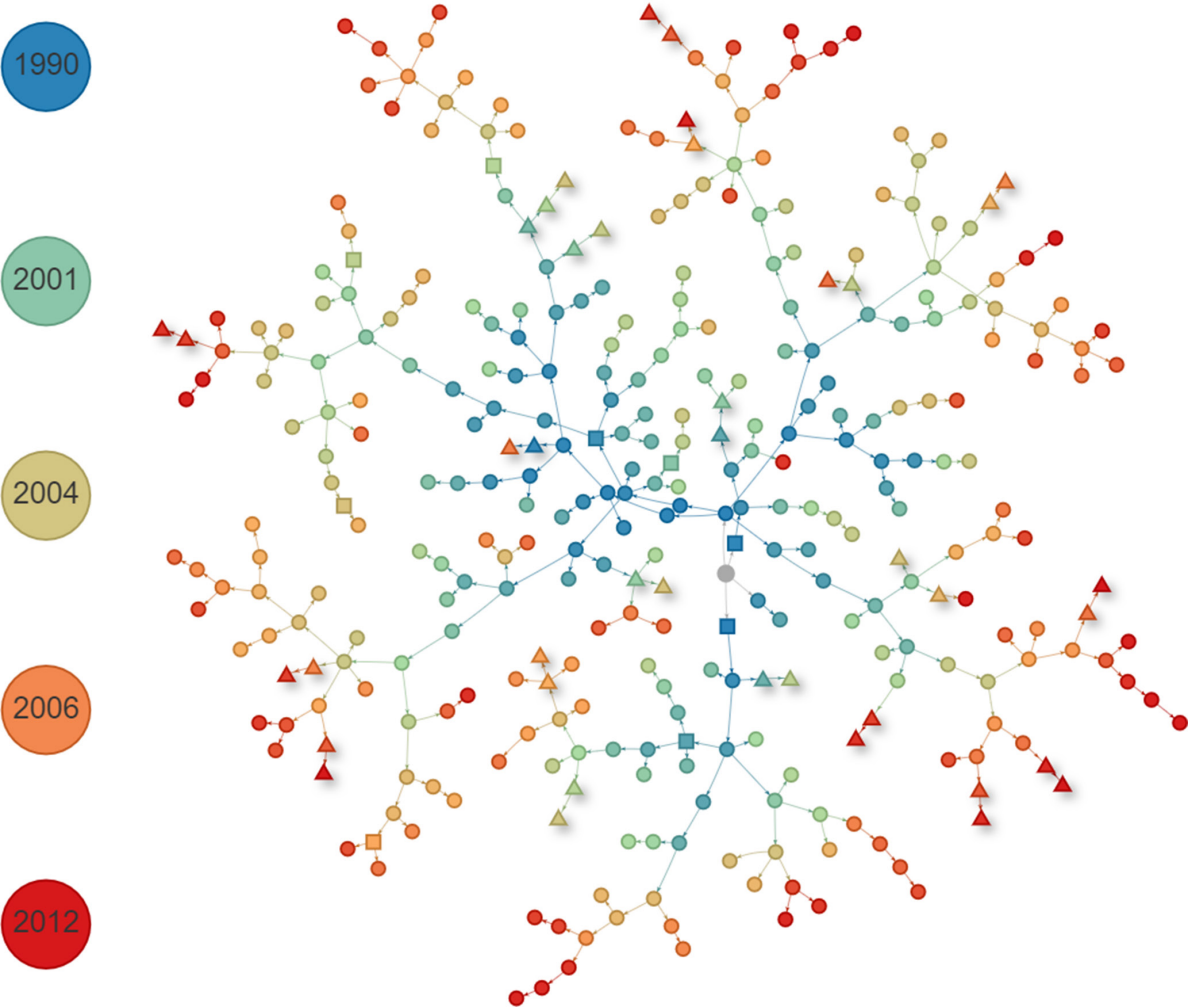
MAP transmission tree of the combined TransPhylo posterior. Nodes (hosts) are coloured by time of infection, with the initial infection coloured in grey. Sampled cases are shown as circles, unsampled cases are shown as squares, and those cases identified as transmission pairs in over 50 % of posterior transmission are shown as triangles. Note that it is not guaranteed that all such transmission pairs will occur as a pair in the MAP tree; here, one identified pair is not in the MAP tree. Shorter edge lengths denote smaller SNP distances.

In order to have a picture of the overall transmission network, we show in Fig. 4 the maximum a posteriori (MAP) transmission tree from the combined TransPhylo posterior. Of all transmission trees sampled, this is the one with the highest posterior probability. One advantage of TransPhylo is its ability to model unsampled cases and estimate their numbers; here, we estimated a mean of 29 unsampled cases (Fig. S3) compared to 329 sampled, resulting in a relatively high case finding rate of 91.2 %. This somewhat reflects our beta (8,2) prior sampling probability which has a mean of 0.8. Fig. S4 shows the MCMC trace plot of the model parameters, which are shared between all 50 simultaneously inferred transmission trees. We discarded the first 50 % of transmission trees as burn-in (to be confident that the MCMC algorithm had reached equilibrium) and sampled only every 50th tree to reduce correlation between successive samples. The final 50 % of the transmission trees (sampled every 50 iterations) corresponding to each of the 50 timed phylogenetic trees were joined together in a combined posterior [of size (10^5^/50)×0.5×50=50 000 trees] that was used for downstream analysis. We calculated the ESS (with an auto-correlation threshold of 0.05) of *r* to be 880 and of π to be 51, both above the usually accepted minimum size of 30. Because they share parameters, the mean of *r*, or equivalently *R*_0_, for any timed tree is 1.09; and the mean sampling probability π is 0.849. Recall that the within-host coalescent time unit (*N_e_g*) was fixed to be 0.27 (100/365).

Investigating the sensitivity of the results to our prior assumptions (see Table S1) revealed that the results are robust to changes in the prior for *N*_*e*_ *g*, as well as *r* being robust to changes in the generation time and sampling time. Outcomes involving sampling of individuals, in particular the sampling proportion π and accordingly the number of credible, sampled transmission pairs, were quite strongly influenced by the generation time and sampling time priors. However, the priors selected in our main analysis are consistent with those used in other analyses of TB outbreaks in well-resourced settings, and do allow for considerable variability in the generation and sampling times.

In contrast to credible TB infectors (see Methods), we sought credible transmission pairs, namely transmission pairs from individual *i* to *j* that have posterior probability greater than 0.5. There are 21 such transmission pairs: 9 with no SNPs, 7 with 1 SNP, 3 with 2 SNPs and 2 with 3 SNPs between the host isolates. We identified no transmission pairs with posterior probability greater than 0.5 in which the infector is unsampled. Previous analysis of this outbreak (see figure 3 in the work by Casali *et al*. [6]) using WGS data identified 5 transmission events (compared to 21 here), though considerable uncertainty remains. There was a maximum of four SNPs in two of the transmission events suggested by WGS in the work by Casali *et al*. [6], in contrast to a maximum of three SNPs in the transmission events identified by our approach. In the 21 pairs we identified, there are 9 pairs where both individuals were treated in the same hospital, 11 pairs where both were of the same ethnicity, 6 pairs where both had been drug users and 5 pairs where both had links with prison. We also note that there is one pair that simultaneously shared all these attributes. One of our identified pairs is in agreement with known reported contacts, but contact data are not available for the majority of our cases.

Even if highly likely pairs are not revealed by WGS data, we can interrogate the Bayesian transmission trees to obtain information that can be useful in outbreak control and case finding. In particular, we explored the relationship between host covariate data and whether hosts are inferred to have infected, or been infected by, unsampled cases.

**Fig. 5.**
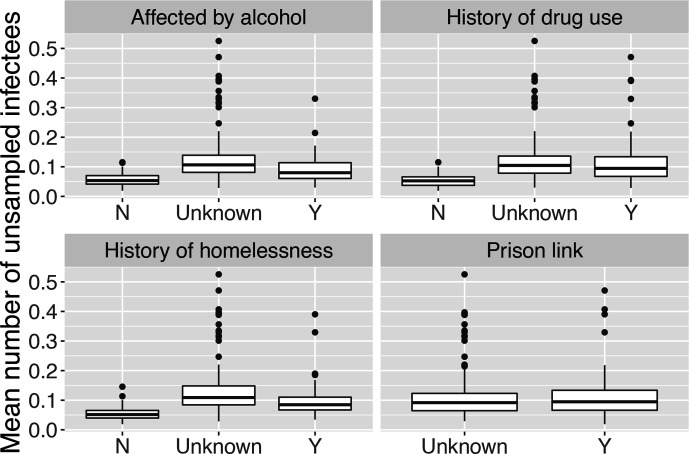
Mean number of unsampled infectees of hosts in different categories defined by four covariates, conditioned on the host infecting at least one other host in the posterior transmission trees. N, No; Y, yes.

**Fig. 6.**
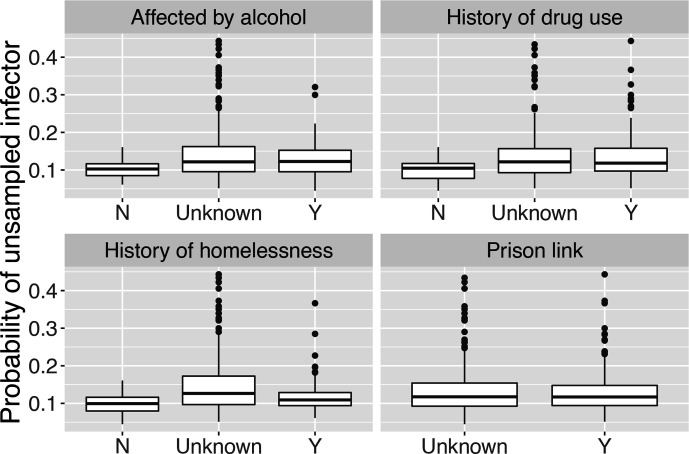
Probability of a host having unsampled infector in different categories defined by four covariates, conditioned on the host not being the index case in the posterior transmission trees. N, No; Y, yes.

Because a host can have many infectees but can only have one infector, we computed the mean number of unsampled infectees over the set of posterior transmission trees in which the host infects at least one other host, and the probability of having an unsampled infector, conditioned on the host not being the index case. We grouped the estimates by four covariates, shown in Figs 5 and 6. We found that an individual tended to infect more unsampled cases if they had been affected by alcohol or drugs, or had a history of homelessness. Based on our data, we cannot conclude whether having a prison link is connected to having more unsampled infectees, because the categories in our prison data are ‘yes’ or ‘NA’ (unknown). The plot of the probability that an individual’s infector is unsampled shows a similar pattern, i.e. an individual is more likely to have an unsampled infector if he or she has used drugs or alcohol or has been homeless, though the distinction is less pronounced.

Our outbreak reconstruction with WGS data can also help interrogate the timing of transmission and sampling in reconstructions consistent with genomic data [27], despite the fact that individual transmission events and their timing is uncertain. The relationship between posterior times of infection and times of sampling is shown in Fig. 3(a). There is an approximately 2 year gap between becoming infected and getting sampled (Fig. 3b). Sensitivity analysis (see Table S1) revealed that this is not particularly driven by the generation time and sampling time priors.

Fig. 3(c) shows how the estimated generation time varies over time. A large proportion of cases had a generation time below 2 years, consistent with previous estimates in similar settings and in this outbreak [8, 23, 27, 28] The estimated mean generation time was lowest in and around 2004. However, not all posterior trees support the assumption that a given individual has infected someone else; in other words, there could be no transmission events between infection and sampling. Each point in Fig. 3(c) is coloured by the probability of the corresponding host ever infecting others. We see that many cases infected recently have a low probability of having infected others and, even if they do, the generation times tend to be short. This is in part due to censoring, as the sampling period ended. However, there were quite a few early cases that are very likely to be transmitters, and their generation times were often long (over 3 years). We note that due to the selection of closely related isolates for inclusion in the study, individuals who reactivated with TB strains that are not considered part of the outbreak are not shown here, and neither are infected individuals who did not become symptomatic before sampling ended. The times to sampling and to onward infection events reflect the censoring and case selection processes.

We sought to relate the covariate data to two outcomes from the outbreak reconstruction: whether an individual is likely to have transmitted TB, and whether an individual has a relatively long or short generation time (time between becoming infected and infecting others). Table 1 shows the aggregated performance of the unoptimized random forest classifiers in a confusion matrix. This compares the predictions from the random forest classifier to the ground truth (which here is assumed from TransPhylo as the ground truth is unknown). In the first task (identifying likely transmitters), we obtained an accuracy of 0.71, precision 0.72 and recall 0.87. A high recall rate is often desirable, because it means that the probability of detecting cases that have infected others will be high. In this case, we obtained a false positive rate of 0.28, which means that one in three or four predicted positives will likely be a false alarm. This could still be helpful to case finding, as overall TB prevalence is low and so false positives may not be a significant burden. Fig. S5(a) shows the receiver operating characteristic (ROC) curve for the first task, with an area under curve (AUC) of 0.72.

**Table 1.** Confusion matrices of the random forest classifier on the validation set Classifications: (a) credible transmitter status, True (T) or False (F); (b) long (L) and short (S) generation times.

(a) Credible transmitters			(b) Generation times		
Actual		Actual	
	F	T		S	L
**Pred F**	56	27	**Pred S**	262	63
**T**	68	178	**L**	3	1

**Fig. 7.**
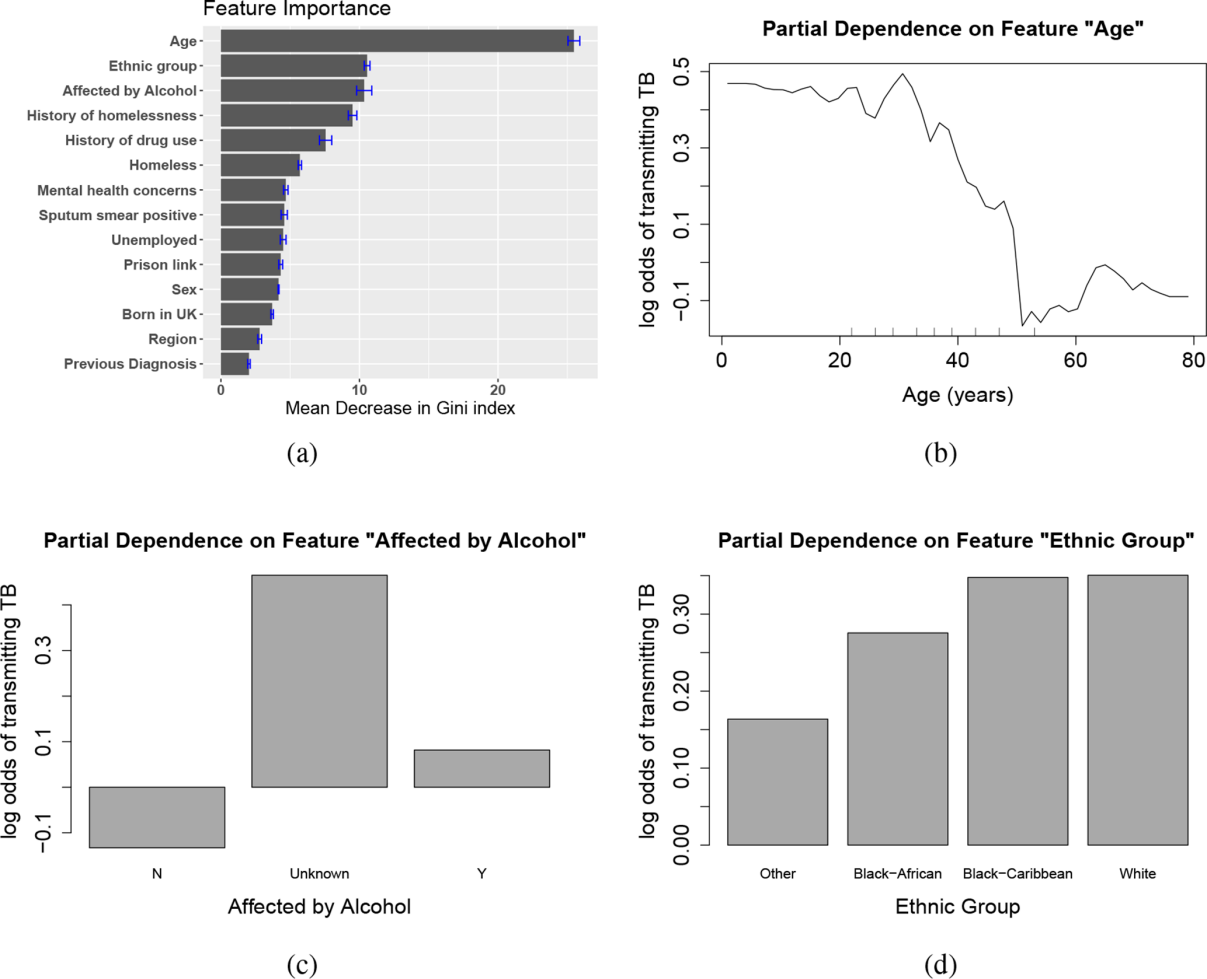
(a) Feature importance plot of the random forest model for classifying whether a host has transmitted TB to others. Importance is measured by the mean decrease in Gini index from splitting on the variable. The error bars are the standard errors of the importance measure on five imputed datasets. (b–d) Partial dependence plots for age, alcoholism and ethnic group. These plot the variable of interest against the log-odds of transmission on a grid of values (age), or discrete categories (alcohol and ethnic group), by marginalizing, or integrating out, all other covariates. N, No; Y, yes.

Fig. 7(a) shows feature importance from the random forest classifier. The importance of a variable is measured by the mean decrease of node impurity, in this case the Gini index, from splitting on that variable in the decision tree. If splitting on variable *A* reduces misclassification more than splitting on variable *B*, then *A* is considered to be more important than *B*. We found that the age of the patient is the most important variable by this measure, outweighing the other variables by a substantial margin. The importance findings may be interesting to epidemiologists, offering insights into variables that may affect the likelihood of transmission. However, we should not be too confident, because the classifier’s performance is not particularly strong (although it is better than random guessing). Including additional covariates may improve classification. Our machine-learning results also suggest that sputum smear status is not particularly important in predicting whether an individual transmitted TB in this outbreak. This may be explained by the fact that concentrated smear testing usually performed in higher-income countries is not as good a marker of infectivity as other approaches.

Partial dependence plots can be used to visualize the marginal effect of some predictors on the response variable, by averaging out the effects of all other variables. For non-categorical features, we can explore whether the relationship between a feature and the response is monotonic, linear or otherwise. In Fig. 7(b–d), we show the partial dependence on age, alcoholism and ethnic group, the three most important predictors, as log-odds. There is a sharp decrease in the likelihood of transmitting TB (according to the fitted model) after age 40; the odds increase somewhat in people over 60. We also note that the log-odds of transmitting TB is a little higher for those who are affected by alcohol than those who are not. The log-odds is largest if the alcoholism variable’s value is unknown, suggesting that there are more positive (Y; i.e. affected by alcohol) than negative (N) cases among those patients who had not reported their alcoholism history. We also observe that individuals of black Caribbean and white heritage are more likely to appear as credible TB transmitters than other ethnic groups in our inference. Partial dependence plots do not reveal whether feature importance is causal or due to residual confounding, so further investigation would be advised.

We also attempted to predict from the metadata whether an individual would have a long or short generation time. Treating the case where the generation time is more than 2 years as the ‘positive’ category, this classifier does not perform as well as trying to predict whether the host has transmitted TB, using the same set of covariates. The confusion matrix of this classifier is shown in Table 1 in (b), the ROC curve in Fig. S5(b), and the feature importance/partial dependence on variable age in Fig. S6.

## Discussion

We have demonstrated how transmission reconstruction from WGS data can be approached using Bayesian statistical inference of transmission trees, with data from a large TB outbreak in London. We have used a modified version of the TransPhylo approach to simultaneously infer transmission events on multiple trees, sharing parameters between them. This allows us to incorporate tree uncertainty into the transmission inference. The ways in which individuals live, work and interact is one of the driving forces for TB transmission [29]. Understanding the relationship between the covariates and transmission gives us insights into factors driving TB transmission, and could provide guidance on effective control mechanisms to public-health authorities.

Although WGS can be insufficient to resolve transmission chains due to lack of detectable variation between cases [5, 6], our statistical approach can refine the analysis usually used in outbreak investigations. We identified more transmission events with reasonable confidence than those that were suggested directly by the data [6]. When patient-level covariates such as demographic and clinical data are available, machine-learning algorithms can be used to predict individual-level variables (i.e. credible transmitter status) derived from transmission reconstruction, providing a means to assess the importance of the covariates for these quantities.

With the move to routine WGS of all TB isolates by Public Health England, it is important to understand the role WGS data can play in outbreak investigations and in understanding transmission. With current sequencing technology and variant-calling pipelines, WGS data may contain insufficient variation to reconstruct individual transmission events with high confidence. It may be that variation simply does not occur rapidly enough in TB to obtain much more information about direct transmission, making the development of approaches to better integrate additional epidemiological data very important [5]. However, sequence data can still contribute to epidemiological analysis through the kind of integrative analysis we have done here, as well as through refuting putative direct-transmission events when the relevant isolates are very distinct genomically. It is possible that new longer-read technologies and improved variant calling may ultimately allow us to capture additional variation occurring in repeat regions and hyper-variable regions, or variation due to insertions and deletions; this would likely be helpful in epidemiological investigations of TB outbreaks in a range of settings.

Our approach has some significant limitations. It is a three-stage approach: reconstruction of timed phylogenetic trees, transmission analysis, followed by machine learning to connect the demographic and clinical data to the transmission analysis. While we have made efforts to take uncertainty into account at each stage by, for example, simultaneously analysing 50 posterior timed phylogenetic trees, joint estimation of the transmission trees and phylogenetic trees together might be preferable if it could be done in a practical way. There would also be advantages to developing statistical and modelling tools to directly (and simultaneously with the phylogeny and transmission trees) estimate the contributions of each covariate to transmissibility, speed of progression of disease and other factors. Instead, here we assumed a ground truth, which was in fact estimated with TransPhylo. We were also limited by considerable amounts of missing data for several covariates including alcohol, drug use and homelessness. Developing the appropriate inference tools would require overcoming the challenge of handling unsampled cases (and the unknown cases they may have infected, and so on) despite the unknown covariate data for the unknown cases. Currently, the mathematics at the heart of TransPhylo does not naturally allow for a likelihood model that extends in this way.

It would additionally help to analyse sequence data together with outbreak control efforts in real time [30, 31]. In TB, with outbreaks lasting years, this is very feasible. Results could inform the outbreak investigation by directing attention towards individuals without a probable infector (and, thus, a likely contact of an unknown case), by informing public-health bodies as to how quickly cases need to be found to interrupt transmission and towards communities or sub-groups with higher numbers of estimated unsampled cases nearby in the transmission tree. To take these actions would require relatively rapid WGS and analyses, but this is now increasingly feasible [32]. WGS data can readily be used to refute transmission events, and routine sequencing has the potential to lead to dramatic improvements in understanding and treating resistant disease, particularly if genome-based resistance predictions can be made quickly enough to inform treatment [32].

One recurring message [5, 6, 8] is that WGS data alone are likely to be insufficient for reconstructing individual transmission events; however, our statistical approach can improve the analysis of WGS data together with covariates, and uncover patterns of transmission. Multiple data sources are required to obtain the best possible understanding of transmission events and transmission patterns. At least with current sequencing and bioinformatics pipelines, clinical, contact, epidemiological and demographic data cannot be replaced with sequencing even though WGS data can have a significant role to play.

## Supplementary Data

Supplementary material 1
