# Supplementary material for: Transmission analysis of a large tuberculosis outbreak in London: a mathematical modelling study using genomic data
Source: Microb Genom. 2020 Nov 11;6(11):mgen000450. doi: 10.1099/mgen.0.000450 (PMC7725332; doi:10.1099/mgen.0.000450)
Supplement: Supplementary material 1 [file mgen-6-450-s001.pdf]

# Supplementary Materials: Transmission analysis of a large tuberculosis outbreak in London: a mathematical modelling study using genomic data

Figure S1 compares the root-to-tip genetic distance of the isolates to their sampling time. Figure S4 shows the trajectories of model parameters during the TransPhylo MCMC run, and Figure S2 is an MDS plot of (a sample from) the posterior collection of phylogenetic trees for 4 independent BEAST chains, through which we find evidence of a unimodal posterior.

Table S1 shows the results of a sensitivity analysis to a number of the model priors. We assess the impact on several key outcomes of varying the mean generation time, the mean sampling time and the within-host coalescent time unit $N_e g$. We vary each quantity by $\pm 50\%$ compared to the main analysis, to explore the result of a significant change. We find that all measured outcomes are fairly robust to changes in $N_e g$. Changes to the assumed mean generation time and sampling time can have considerable impact on the outcomes, particularly, as would be expected, on those measures related to sampling of individuals: the sampling proportion, the number of credible sampled transmission pairs. Notably, both the reproductive number $r$ and the average time from infection to sampling are relatively unchanged by changes in the priors.

Figure S3 shows the estimated number of unsampled cases, and Figure S5 shows the receiver-operator characteristic curves for the classification tasks. A random classifier would have an area under the curve (AUC) on these plots of 0.5. Our results suggest that the covariates are better predictors of whether an individual transmits TB than of whether an individual has a longer-than-usual generation time, but we note that these results use the TransPhylo estimates in place of the true status (infector; generation time), as the truth is not known. Figure S6 shows the feature importance and partial dependence on feature age for the second machine learning task: classifying long/short generation time of hosts.

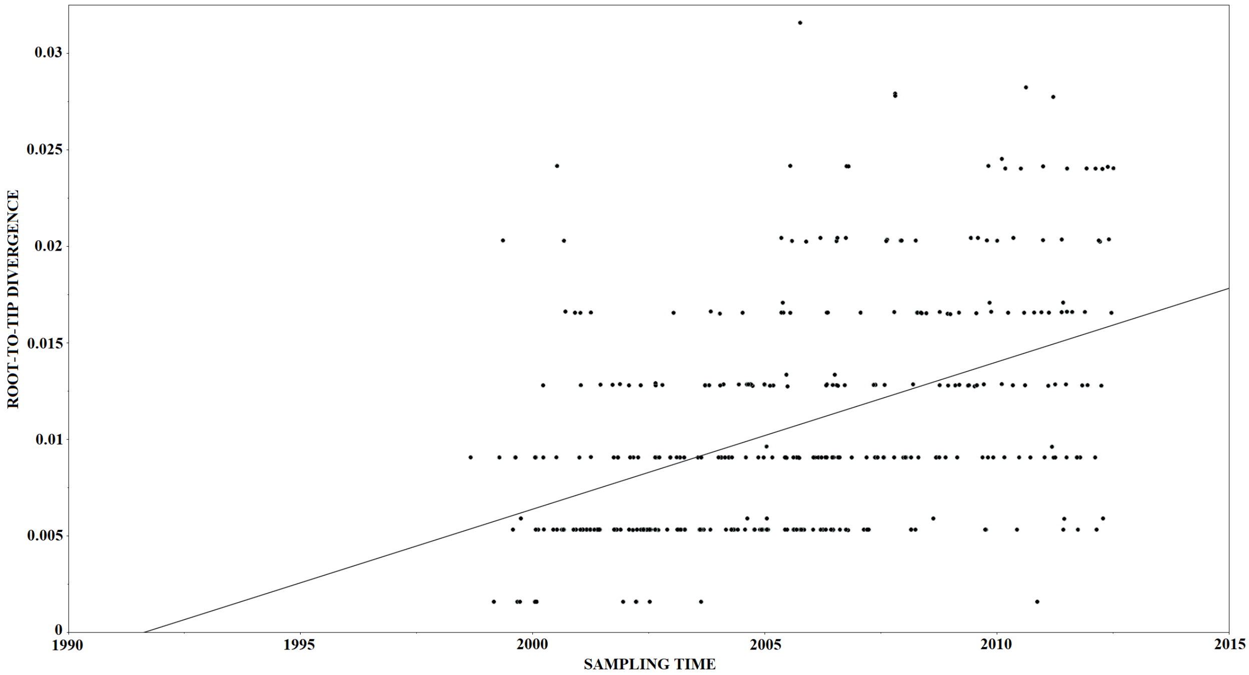


Figure S1: Plot of phylogenetic root-to-tip genetic distance relative to sampling time using TempEst. Each dot represents one sample per data set.

| | Main analysis | gen. time $-50\%$ | gen. time $+50\%$ | sampling time $-50\%$ | sampling time $+50\%$ | $N_e g$ $-50\%$ | $N_e g$ $+50\%$ |
|---|---|---|---|---|---|---|---|
| $r$ | 1.087 | 1.053 | 0.906 | 0.993 | 1.118 | 1.083 | 1.089 |
| $\pi$ | 0.849 | 0.228 | 0.985 | 0.411 | 0.980 | 0.817 | 0.860 |
| Unsampled cases | 29 | 376 | 2 | 190 | 3 | 35 | 26 |
| Infection to sampling (years) | 2.40 | 1.85 | 2.43 | 1.3 | 2.69 | 2.5 | 2.302 |
| Credible transmission pairs | 21 | 2 | 39 | 0 | 51 | 22 | 34 |

Table S1: Analysis of sensitivity to model assumptions. In each case, we adjust the mean of the generation time/time to sampling/parameter $N_e g$ priors by the amount shown, whilst keeping the variance constant. Across these different assumptions, we compare mean estimates of parameters $r$ and $\pi$, the median number of estimated unsampled cases, the mean time from infection to sampling and the number of identified transmission pairs with probability $> 0.5$. In the main analysis, mean generation time$= 3.25$, mean time to sampling$= 6.6$, and $N_e g = 0.27$.

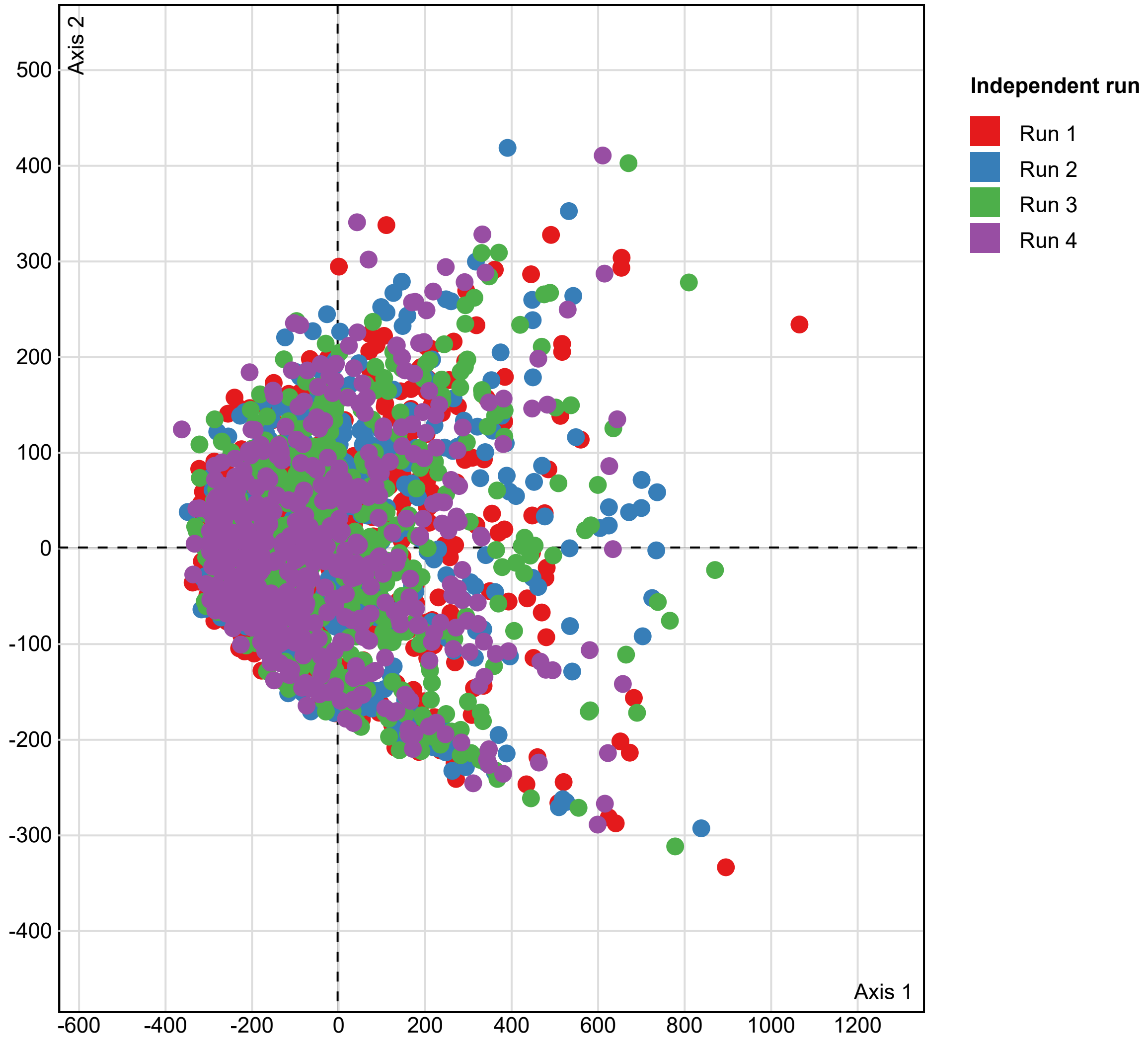


Figure S2: Scatterplot of the Multidimensional Scaling (MDS) output from treespace, for 4 independent BEAST chains. This is a 2D representation of the space of 500 phylogenetic trees sampled at random from the posterior of each of 4 runs - each point represents a single tree.

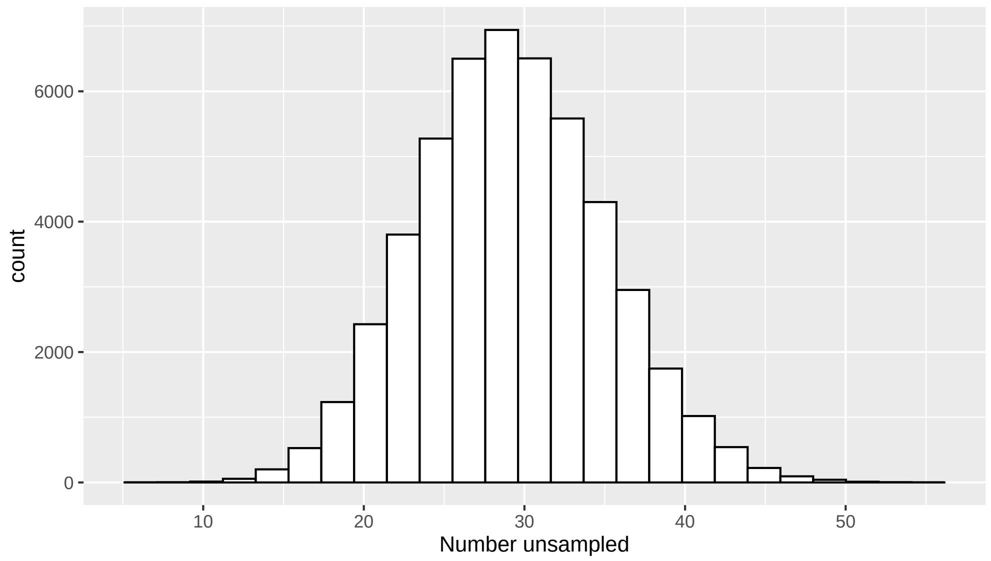


Figure S3: Histogram of number of unsampled cases, generated from the combined posterior transmission trees.

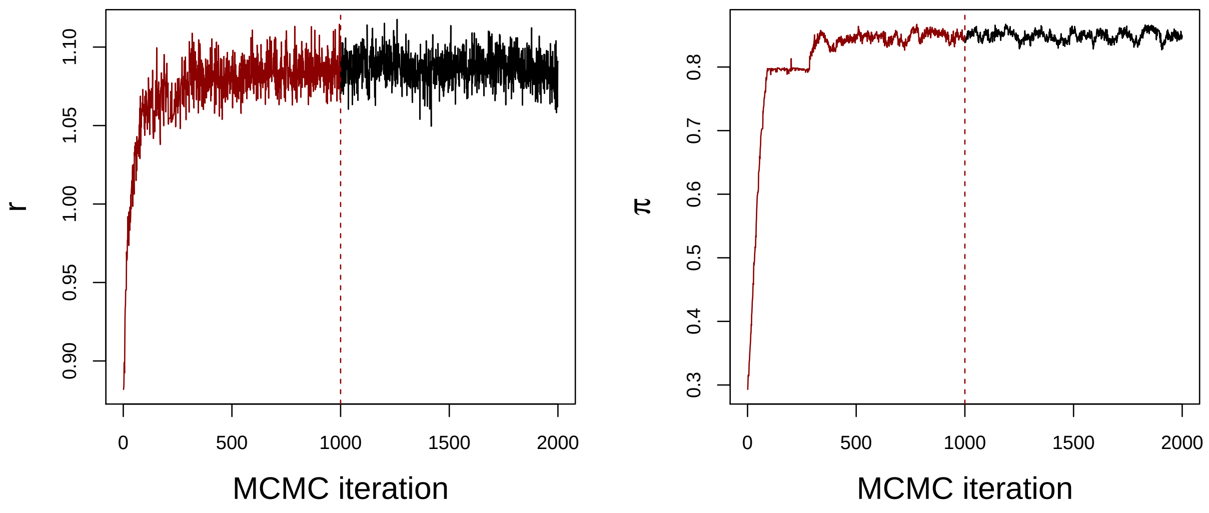


Figure S4: Trace plot of model parameters $r$, the first parameter of negative binomial offspring distribution or equivalently the basic reproduction number, and the sampling probability $\pi$. Burn-in is coloured red.

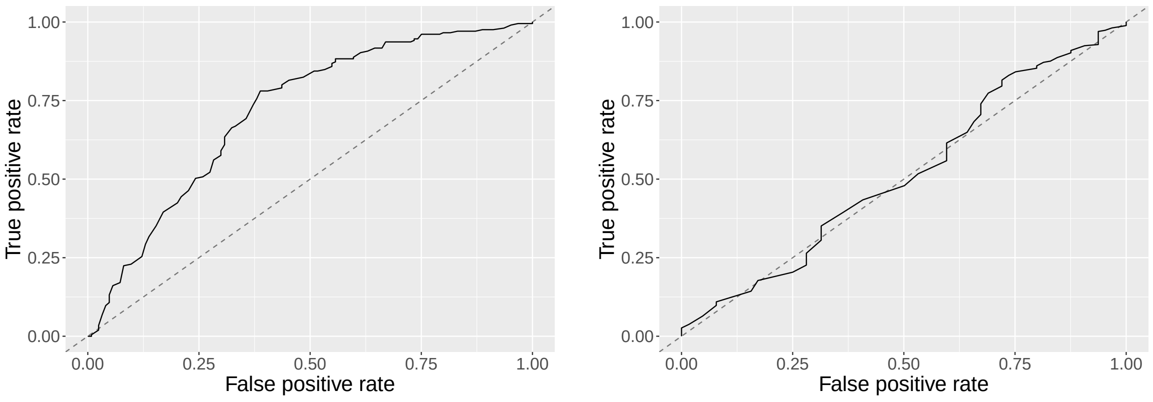


(a) (b)

Figure S5: ROC curves illustrating the overall performance of the classifiers. A random classifier is expected to have an AUC (area under the curve) of 0.5 and perfect classification, with no false positives or false negatives, achieves an AUC of 1. (a): Task one: predicting if a host has transmitted TB. (b) Task two: predicting if a host has over 2 years generation time.

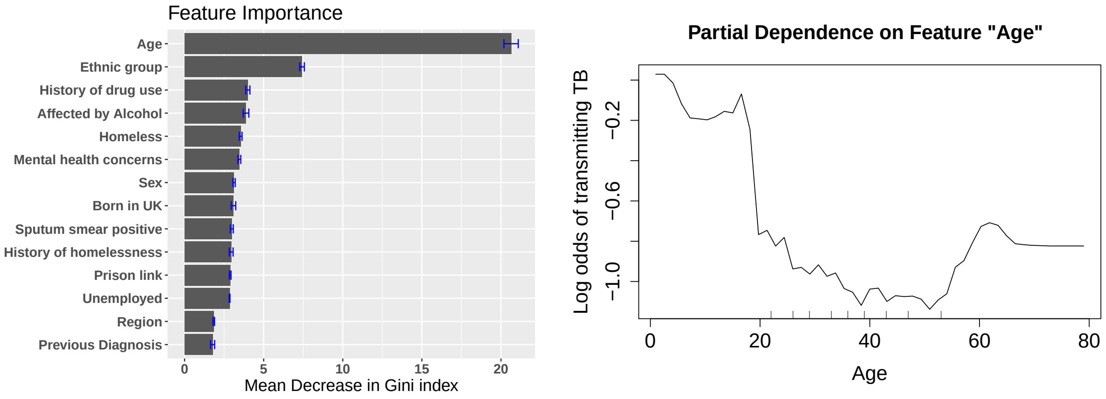


(a) (b)

Figure S6: (a): Feature importance plot of the random forest model for classifying whether a host has generation time greater than 2 years. Importance is measured by the mean decrease in Gini index from splitting on the variable. The error bar is the standard error of the importance measure on 5 imputed datasets. (b): Partial dependence plot for age variable.
